# Cross metathesis of unsaturated epoxides for the synthesis of polyfunctional building blocks

**DOI:** 10.3762/bjoc.11.201

**Published:** 2015-10-08

**Authors:** Meriem K Abderrezak, Kristýna Šichová, Nancy Dominguez-Boblett, Antoine Dupé, Zahia Kabouche, Christian Bruneau, Cédric Fischmeister

**Affiliations:** 1 Université Frères Mentouri Constantine, Department of Chemistry, Laboratory of Therapeutic Substances Obtention (LOST), Chaabet Ersas Campus, 25000 Constantine, Algeria; 2 UMR6226 CNRS, Institut des Sciences Chimiques de Rennes, Université de Rennes 1, Organometallics: Materials and Catalysis, Centre for Catalysis and Green Chemistry, Campus de Beaulieu, 35042 Rennes Cedex, France; 3 Charles University in Prague, Faculty of Science, Department of Physical and Macromolecular Chemistry, Hlavova 2030, CZ-128 40 Prague, Czech Republic; 4 Faculty of Chemistry, University of Seville, E-41012 Seville, Spain

**Keywords:** cross metathesis, epoxide, ruthenium catalysts, tandem reactions

## Abstract

The cross metathesis of 1,2-epoxy-5-hexene (**1**) with methyl acrylate and acrylonitrile was investigated as an entry to the synthesis of polyfunctional compounds. The resulting cross metathesis products were hydrogenated in a tandem fashion employing the residual ruthenium from the metathesis step as the hydrogenation catalyst. Interestingly, the epoxide ring remained unreactive toward this hydrogenation method. The saturated compound resulting from the cross metathesis of **1** with methyl acrylate was transformed by means of nucleophilic ring-opening of the epoxide to furnish a diol, an alkoxy alcohol and an amino alcohol in high yields.

## Introduction

Catalytic carbon–carbon double bond transformations by olefin metathesis have significantly impacted organic and polymer synthesis over the last two decades [1–3]. If early works focused on ring-closing metathesis and ring-opening metathesis polymerization, progresses in catalysts performances [4–5] and selectivity have enabled the achievement of more challenging transformations such as cross metathesis reactions [6], stereoselective transformations [7] including the selective synthesis of *Z*-olefins [8–11]. Recently, the cross metathesis of renewable compounds with electron-deficient olefins was developed as a straightforward way for the synthesis of difunctional compounds suitable for polymer syntheses [12–13], fine chemicals [14–17], or as key synthetic tool in multistep syntheses of complex molecules [18–21]. Cross metathesis with functional olefins is of great interest as it offers the possibility for post-transformation of the functional group. For example we have shown that cross metathesis with acrylonitrile run in a tandem fashion with hydrogenation delivered amine derivatives [22] whereas the tandem cross metathesis/hydrogenation with acrolein delivered the corresponding alcohols [23–24]. Nice examples of cross metathesis/non-metathesis sequences have also been reported by Andrade in 2011 [25].

In this article we present our results aimed at extending the scope of sequential transformations including cross metathesis to the synthesis of trifunctional compounds. Several examples involving the cross metathesis of a commercially available epoxide-containing olefin with methyl acrylate and acrylonitrile and their subsequent transformations leading to multifunctional building blocks are reported.

## Results and Discussion

**Scheme 1:**
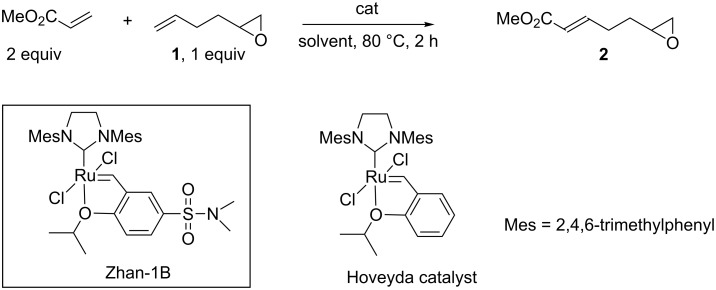
Cross metathesis of **1** with methyl acrylate.

Cross metathesis reactions involving electron-deficient olefins are generally challenging transformations as they are substrate-dependent and therefore require optimization of experimental parameters. For instance, while cross metathesis with methyl acrylate turns out to be a rather straightforward transformation, cross metatheses with acrylonitrile, acrylamides or acrolein are much more demanding transformations [13–14,24]. We have investigated the reactivity of 1,2-epoxy-5-hexene (**1**) with methyl acrylate and acrylonitrile and further exploited the versatility of the epoxide ring to prepare trifunctional molecules by ring opening of the epoxide. To date, **1** has been scarcely used in olefin cross metathesis transformations. In some examples, Grela used **1** as a test substrate to evaluate the efficiency of new catalysts [26], and Cossy prepared vinyl functionalized oxazoles [27]. To our knowledge, the cross metathesis of **1** with electron-deficient olefins has not been reported. The cross metathesis of **1** with methyl acrylate was thus investigated under various conditions of solvents, catalysts and concentration (Scheme 1). As required in cross metathesis reactions of electron-deficient olefins, an excess of methyl acrylate was employed and a temperature of 80 °C was necessary to ensure high conversion. Reactions were carried out in dimethyl carbonate (DMC), a solvent compatible with ruthenium olefin metathesis catalysts [28] while being much greener than toluene or dichloromethane commonly used in such reactions [29]. Based on our previous results and observations in various cross metathesis reactions, the phosphine-free Hoveyda type second generation Zhan catalyst-1B [30] was selected to conduct this transformation. A recent study by Fogg rationalized the superiority of the Hoveyda catalyst vs the Grubbs catalyst in cross metathesis with acrylates showing that the phosphine could interact with the electron-deficient olefin leading to catalyst decomposition [31].

As observed by us and other groups in cross metathesis involving different substrates, double bond migration side-reactions took place during this transformation. This side reaction could be circumvented using benzoquinone [32] as an additive to decrease the extent of double-bond migration. As depicted in Table 1 (entries 1–4), 10 mol % of benzoquinone were necessary to ensure a limited amount (<10%) of side products resulting from double-bond migration. However, addition of benzoquinone resulted in slower reaction hence a catalyst loading of 2 mol % was necessary to restore full conversion within 2 h (Table 1, entry 4). In this case the product was isolated by distillation [33] in 69% yield as the sole *E*-isomer [34]. The transformation was sensitive to the concentration of the reagents and required a concentration of 0.5 M to operate with full conversion. This characteristic was previously observed in cross metathesis of fatty acid methyl esters with methyl acrylate [13]. Finally, neither toluene as solvent nor Hoveyda 2^nd^ generation catalyst have led to improvements of the reaction performances (Table 1, entries 7 and 8).

**Table 1:** Cross metathesis of **1** with methyl acrylate^a^.

Entry	[**1**] (mol·L^−1^)	Cat. loading (mol %)	BQ^b^ (mol %)	Conv. (%)^c^ (yield %)^d^	% isom.^e^

1	0.5	1	5	100	13
2	0.5	2	5	100	15 (18)^f^
3	0.5	1	10	95	7.6
4	0.5	2	10	100 (69)	7
5	0.25	2	10	95	8
6	1	2	10	100	10
7^g^	0.5	2	10	100	8
8^h^	0.5	2	10	90	11

^a^0.11 mL of **1** (1 mmol), 0.18 mL of methyl acrylate (2 mmol), BQ, DMC, catalyst, 2 h; ^b^benzoquinone; ^c^determined by gas chromatography using dodecane as internal standard; ^d^isolated yield; ^e^determined by gas chromatography as ratio of ((isomerisation products)/(isomerisation products + **2**)) × 100; ^f^reaction performed without benzoquinone; ^g^in toluene; ^h^Hoveyda 2^nd^ gen. catalyst.

**Scheme 2:**
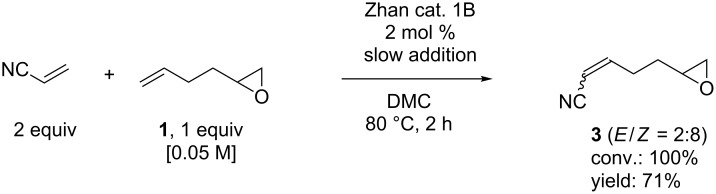
Cross metathesis of **1** with acrylonitrile.

Similarly, the cross metathesis of **1** with acrylonitrile was conducted to furnish the bifunctional derivative **3** in 71% yield as a mixture of stereoisomers. In that case, high conversions and yields could only be obtained by means of slow addition of the catalyst and high dilution (Scheme 2) [13]. As we already observed, [13–14,22] together with other groups, [35–36] in various cross metathesis reactions involving acrylonitrile, the cross metathesis product **3** was obtained as a mixture of *E* (minor) and *Z* (major) stereoisomers.

**Scheme 3:**
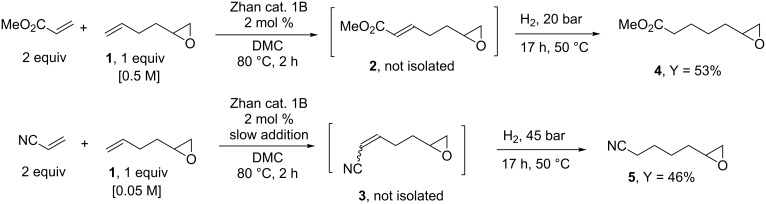
Tandem cross metathesis/hydrogenation.

With these two compounds in hands, we turned our attention to their post-metathesis transformations. First, we looked at the hydrogenation of the carbon–carbon double bond in compounds **2** and **3**. Typically, there are several ways to perform the hydrogenation of a carbon–carbon double bond resulting from a cross metathesis reaction. A possibility consists in the Pd/C catalyzed hydrogenation of the isolated product. This method presents the advantage of being effective at room temperature under a low hydrogen pressure [37–38]. However, such hydrogenations are in general carried out on purified products but more importantly in the present case, such conditions may result in the carbon–carbon double bond hydrogenation accompanied by ring opening of the epoxide leading to a mixture of primary and secondary alcohols [39]. A second and more straightforward method consists in the tandem metathesis/hydrogenation reaction where the residual ruthenium species arising from the metathesis step serve as the hydrogenation catalyst [13,22–24,40]. In general, this protocol requires higher temperature and pressure but it does not need additional costly catalyst and it can be performed without isolation of the intermediate olefin hence saving time and energy-consuming work-up procedures [41]. To the best of our knowledge, such a tandem procedure has not been applied to an epoxide containing olefin. Compound **2** was prepared as described here above (Scheme 1) and the reaction mixture was directly transferred into a high pressure reactor without any work-up. Remarkably, following the hydrogenation step carried out under 20 bar of hydrogen at 50 °C, the ^1^H NMR of the crude reaction mixture revealed the presence of the epoxide moiety without any traces of alcohol. This tandem procedure delivered the saturated compound **4** in a satisfactory 53% yield for two steps (Scheme 3). The tandem cross metathesis of **1** with acrylonitrile followed by hydrogenation of the intermediate compound **3** was conducted similarly. In this case a higher hydrogen pressure (45 bar) was necessary to reduce the carbon–carbon double bond. Nevertheless, under these conditions, the epoxide-containing product **5** was isolated in a satisfactory 46% yield for two steps without any traces of alcohol detected in the crude ^1^H NMR of the reaction.

**Scheme 4:**
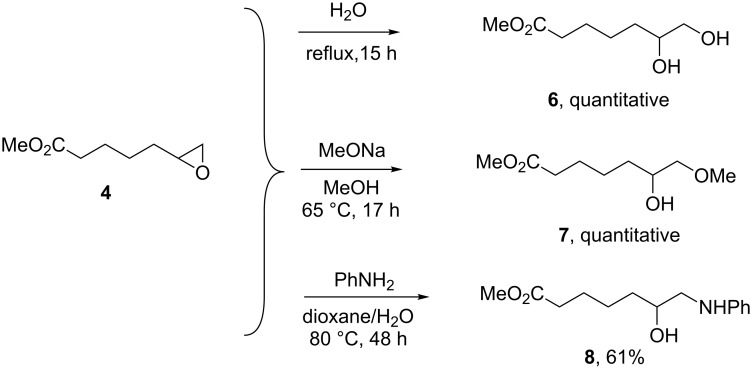
Trifunctional compounds obtained by ring-opening of epoxide **4**.

With this protocol secured, we turned our attention to the synthesis of useful polyfunctional building blocks. Thus far, the post-transformation of the electron- deficient olefin cross metathesis partner has received attention for the synthesis of polymer precursors. For instance, we have reported the reduction of the nitrile functional group into primary amine [22] and the reduction of the formyl group into alcohol [23–24]. Herein, we focused on the post-transformation of **4** by ring-opening of the epoxide moiety. The diol **6**, methoxy alcohol **7** and amino alcohol **8** were thus prepared by reacting **4** with water, sodium methoxide and aniline, respectively (Scheme 4). The synthesis of **6** proceeded cleanly and did not require any purification procedure (see Supporting Information File 1). Similarly, the synthesis of **7** proceeded cleanly and delivered a single regioisomer **7** in quantitative yield. Finally, the amino alcohol **8** was also obtained as a single regioisomer in 61% yield (Scheme 4).

## Conclusion

We have shown through selected examples that cross metathesis of an epoxide containing olefin with electron-deficient olefins constitutes a versatile entry towards trifunctional building blocks by ring-opening of the epoxide. We have shown that the tandem cross metathesis/C=C hydrogenation yielded the hydrogenated compound without altering the epoxide moiety that was further efficiently transformed into a 1,2-diol, a 1,2-alkoxy alcohol and a 1,2-amino alcohol. This strategy opens the way for numerous potential transformations involving the epoxide but also the functional group of the electron-deficient olefin. In particular, lactones should be accessible by intramolecular *trans*-esterification from **6**, **7** and **8**, as well as cyclic amines by intramolecular cyclization involving primary amine resulting from hydrogenation of the nitrile functionality in **5**. All these aspects will be further developed in our group.

## Supporting Information

File 1 Full experimental details and characterizations.
