# Supplementary material for: Cross metathesis of unsaturated epoxides for the synthesis of polyfunctional building blocks
Source: Beilstein J Org Chem. 2015 Oct 8;11:1876–80. doi: 10.3762/bjoc.11.201 (PMC4660967; doi:10.3762/bjoc.11.201)
Supplement: File 1 — Full experimental details and characterizations. [file Beilstein_J_Org_Chem-11-1876-s001.pdf]

Supporting Information

for

Cross metathesis of unsaturated epoxides for the synthesis of polyfunctional building blocks

Meriem K. Abderrezak[1,2], Kristýna Šichová[3,2], Nancy Dominguez-Boblett[4,2], Antoine Dupé[2], Zahia Kabouche[1], Christian Bruneau[2], Cédric Fischmeister*[2]

Address: [1]Université Frères Mentouri Constantine, Department of Chemistry, Laboratory of Therapeutic Substances Obtention (LOST), Chaabet Ersas Campus, 25000 Constantine, Algeria, [2]UMR6226 CNRS, Institut des Sciences Chimiques de Rennes, Université de Rennes1, Organometallics: Materials and Catalysis, Centre for Catalysis and Green Chemistry, Campus de Beaulieu, 35042 Rennes Cedex, France, [3]Charles University in Prague, Faculty of Science, Department of Physical and Macromolecular Chemistry, Hlavova 2030, CZ-128 40 Prague, Czech Republic and [4] Faculty of Chemistry, University of Seville, E-41012 Seville, Spain

Email: Cédric Fischmeister - cedric.fischmeister@univ-rennes1.fr

*Corresponding author

Full experimental details and characterizations

All reactions were carried out under an inert atmosphere of argon using standard Schlenk tube techniques. Dimethyl carbonate (DMC) was distillated under atmospheric pressure and stored over activated 4 Å MS. Other solvents were dried by conventional distillation procedure or using a MBraun SPS apparatus. 1,2-Epoxy-5-hexene (**1**) was purchased from Sigma-Aldrich and used as received. Methyl acrylate was obtained from a commercial source and stored under argon over 4 Å MS. Acrylonitrile was obtained from a commercial source, distilled over P_2O_5 and stored under argon over 4 Å MS. Zhan 1B catalyst was purchased from Strem chemicals. Hoveyda catalyst was purchased from Sigma-Aldrich. The reactions were monitored using a Shimadzu 2014 gas chromatograph equipped with an Equity™ – 1 Fused Silica capillary column (30 m × 0.25 mm × 0.25 μm) and a FID detector. Conversions were determined by internal calibration using dodecane as standard. Proton magnetic resonance (^{1}H NMR) spectra were recorded on a Bruker Avance III 400 MHz spectrometer. ^{1}H and ^{13}C chemical shifts (δ) are reported in ppm vs tetramethylsilane (δ = 0.00 ppm) and determined by reference to the residual non-deuterated solvents (δ = 7.26 for C*H*Cl_3 and 77.0 *C*HCl_3). Coupling constants (*J*) are given in Hertz.

(E)-Methyl 5-(oxiran-2-yl)pent-2-enoate (2)

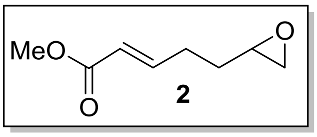

A vacuum-dried Schlenk tube was loaded with 29 mg (2 mol %) of Zhan catalyst-1B, 22 mg (10 mol %) of benzoquinone, 4 mL of dimethyl carbonate and 20 μL of dodecane as GC internal standard. 0.362 mL (4 mmol, 2 equiv) of methyl acrylate and 0.22 mL (2 mmol, 1 equiv.) of 1,2-epoxy-5-hexene (**1**) were then added and the resulting mixture was stirred at 80 °C for 2 h. Solvent evaporation under moderate vacuum (volatile product) followed by distillation afforded 212 mg (yield = 69%) of the desired product obtained as a colourless oil.

^{1}H NMR (400 MHz, $CDCl_3$): δ = 6.96 (dt, 1 H, J = 15.6 Hz, 6.8 Hz, CH), 5.85 (d, 1H, J= 15.6 Hz, CH), 3.71 (s, 3 H, OCH_3), 2.95-2.90 (m, 1 H, CH), 2.78 (m, 1 H, CH), 2.48 (dd, J = 5.0 Hz, 2.7 Hz, 1 H, C*H*), 2.40-2.30 (m, 2 H, CH_2), 1.82-1.70 (m, 1 H, CH), 1.67-1.57 (m, 1 H, CH).

^{13} C NMR (100 MHz, $CDCl_3$): δ = 166.9, 147.8, 121.7, 51.5, 51.4, 47.1, 30.9, 28.6.

HRMS (ESI): [M+Na]+ ($C_8H_{12}O_3Na$); Th: 179.0684, exp: 179.0682

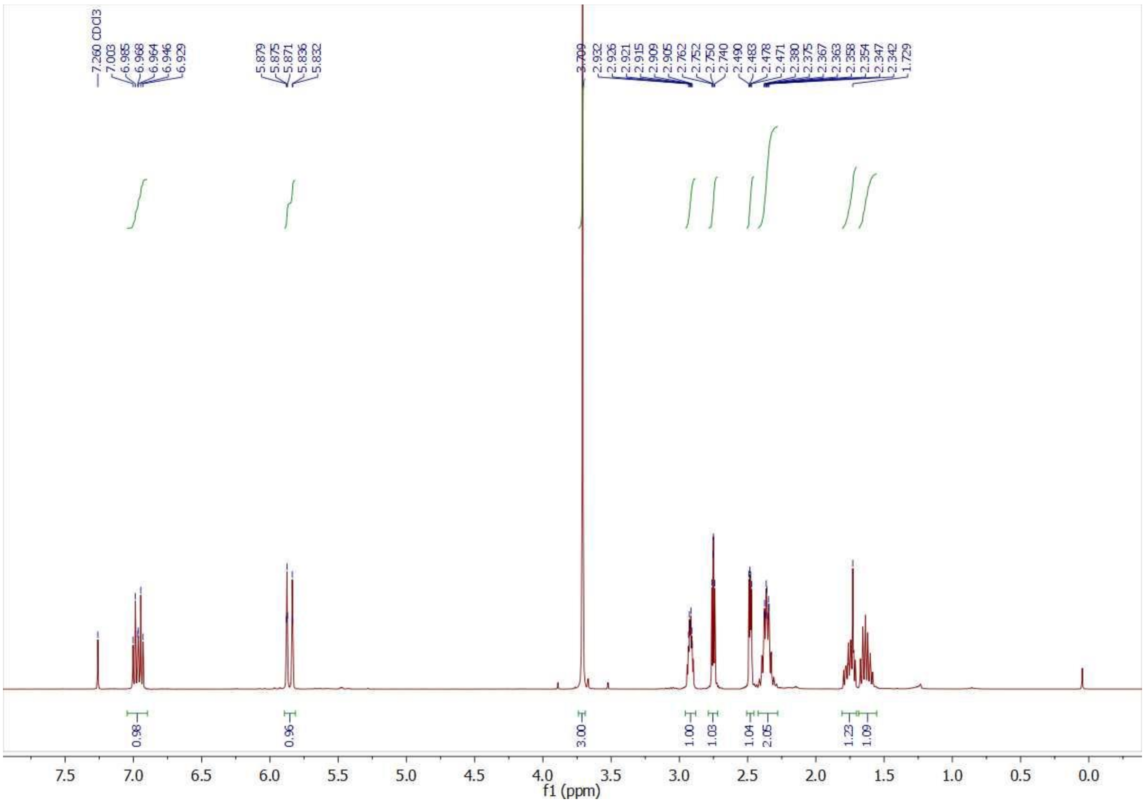

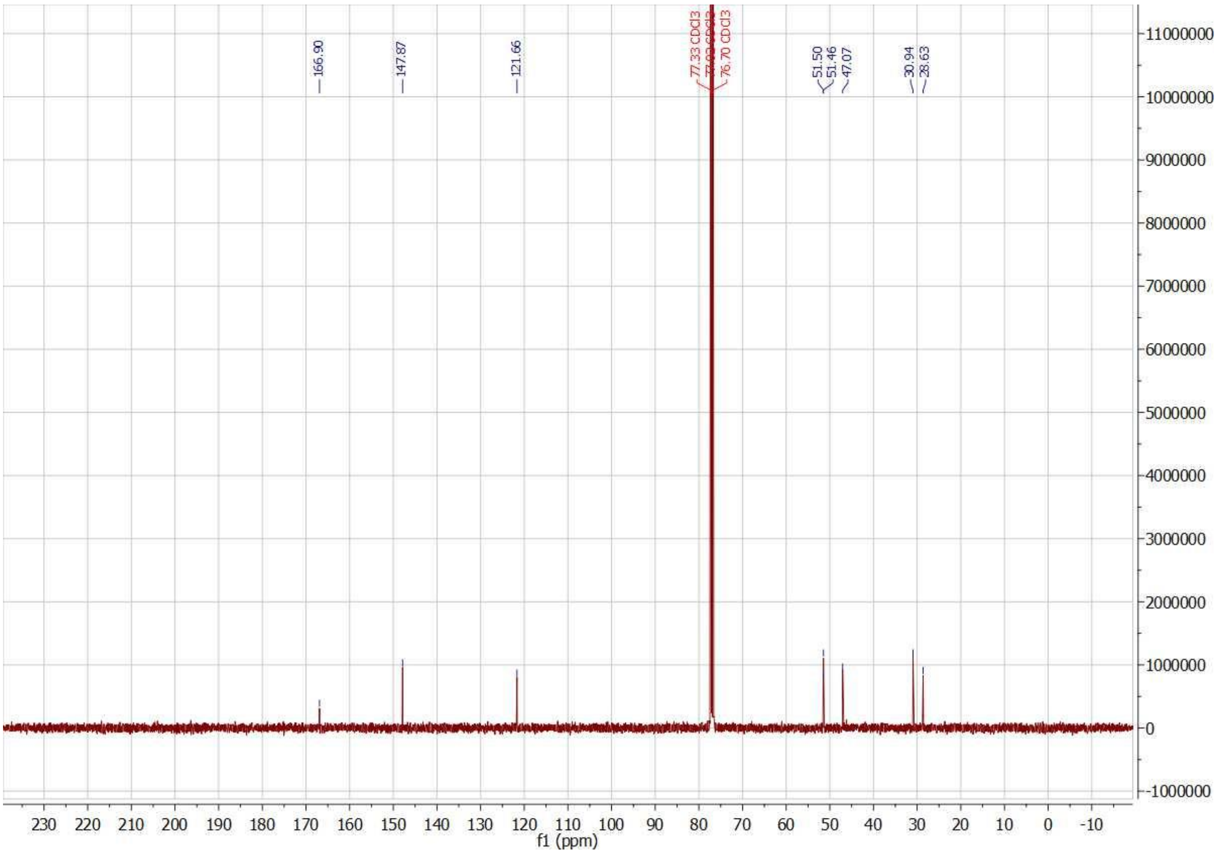

***(E,Z)*-1-Cyano-4-(oxiran-2-yl)but-1-ene (3)**

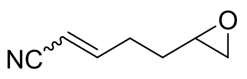

3

A vacuum-dried Schlenk tube was loaded with,11 mg (10 mol %) of benzoquinone, 15 mL of dimethyl carbonate and 20 µL of dodecane as GC internal standard. 0.13 mL (2 mmol, 2 equiv) of acrylonitrile and 0.11 mL (1 mmol, 1 equiv) of 1,2-epoxy-5-hexene (**1**)were then added and the resulting mixture was stirred at 80 °C for 2 h in an oil bath. To this solution was added a solution of 15 mg (2 mol %) of Zhan catalyst-1B disolved in 5 mL of DMC (syringe pump, 2 h addition time). Solvent evaporation under moderate vacuum (volatile product) followed by distillation afforded 88 mg (yield = 71%, colourless oil) of the desired product obtained as mixture of *Z*/*E* stereoisomers (80/20).

^{1}H NMR (400 MHz, $CDCl_3$): δ = 6.74 (dt, J = 16.4, 6.9 Hz, 0.18 H *E*, C*H*), 6.54 (dt, 0.77 H *Z*, J = 10.9, 7.6 Hz, C*H*), 5.4-5.37 (m, 1H *E*+*Z*, C*H*), 2.90-2.96 (m, 1H, C*H*), 2.80-2.75 (m,

1*H*), 2.65-2.55 (m, 1.6 H, CH_2(C*H*=) *E*), 2.51 (m, 1H, C*H*), 2.40 (m, 0.4H, CH_2(C*H*=) *Z*), 1.85-1.75 (m, 1H, C*H*), 1.70-1.60 (m, 1H, C*H*).

^{13}C NMR (100 MHz, $CDCl_3$): δ = 154.5 (CH (*E*)), 153.8 (CH (*Z*)), 117.3 (CN (*E*)), 115.9 (CN (*Z*), 100.8 (CH (*E*)), 100.5 (CH (*Z*)), 51.4 (CH (*Z*)), 51.3 (CH (*E*)), 47.1 (CH_2 (*E*)), 46.9 (CH_2 (*Z*)), 31.2 (CH_2 (*Z*)), 30.7(CH_2 (*E*)), 30.0 (CH_2 (*E*)), 28.6 (CH_2 (*Z*)).

HRMS (ESI): [M+Na]+ (C_7H_9NONa); Th: 146.0581, exp: 148.0583

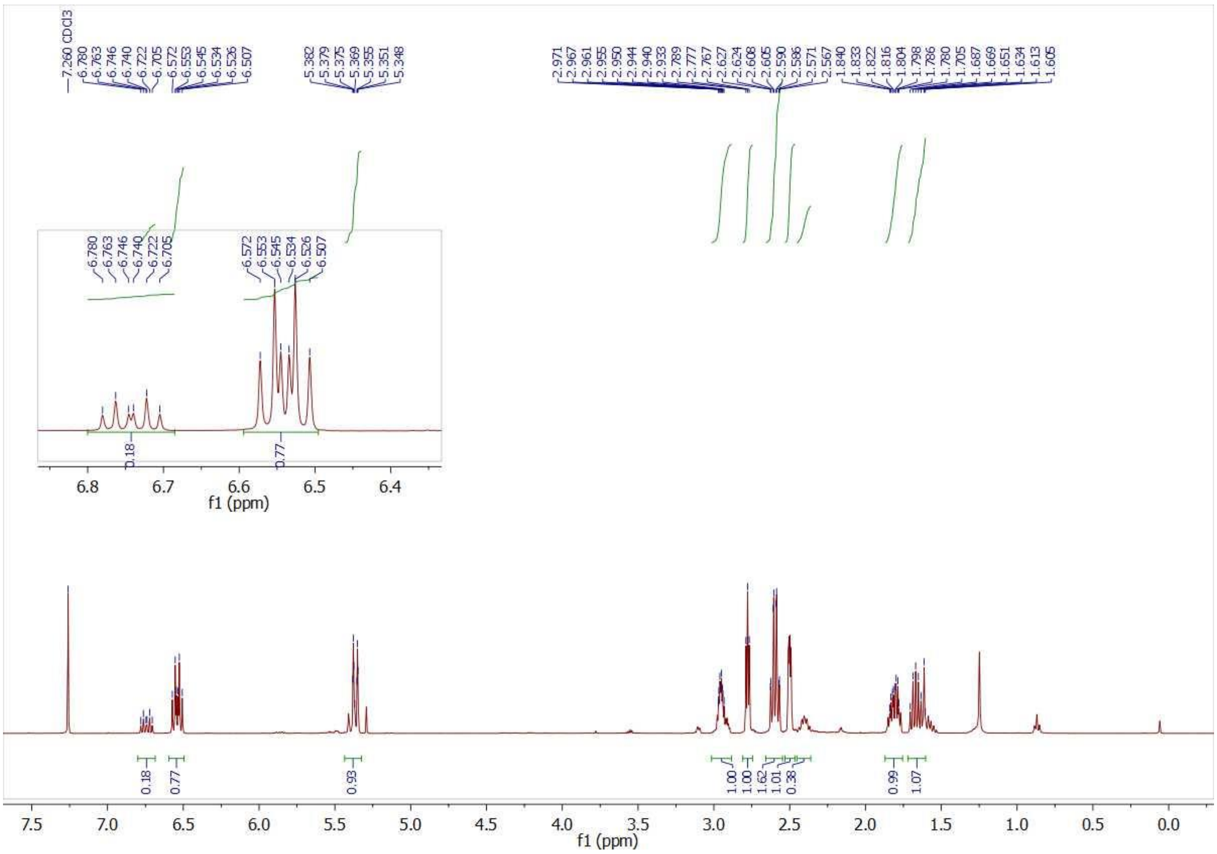

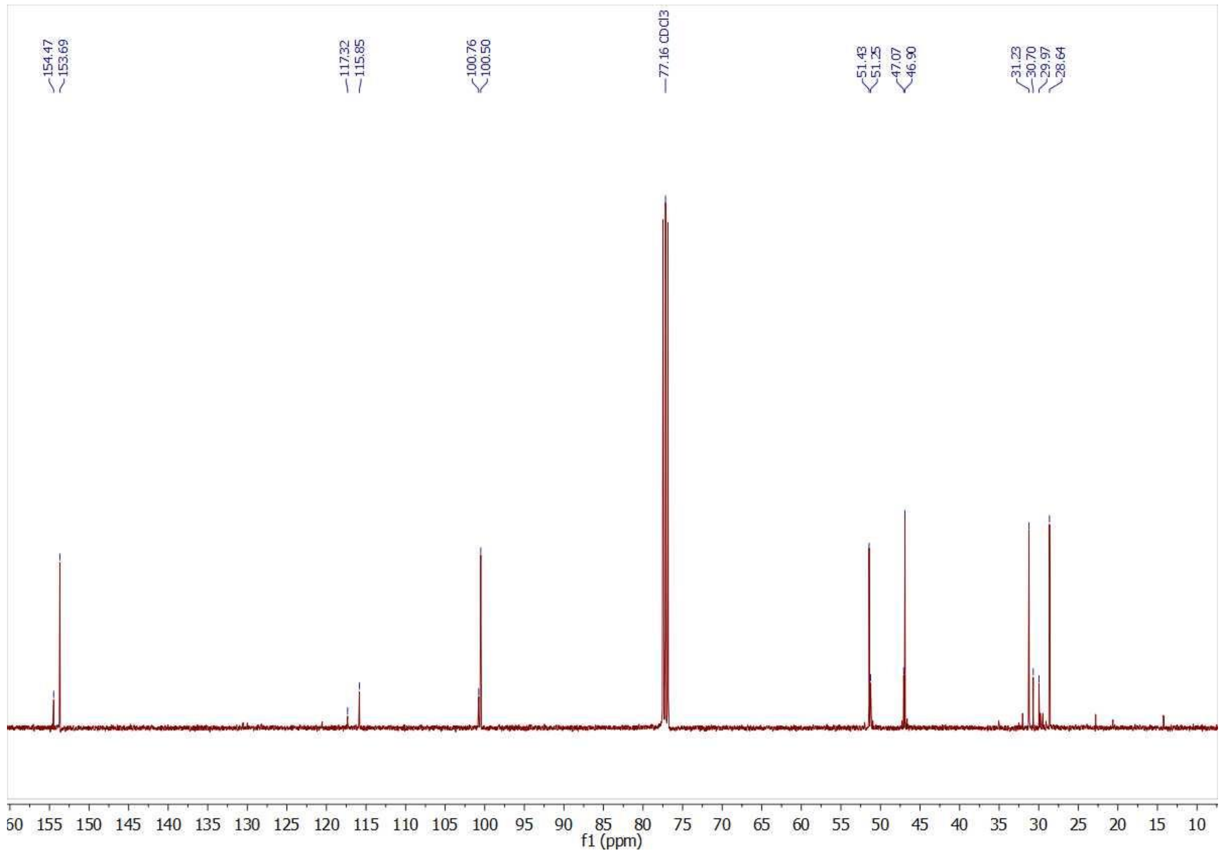
154.47
153.69
117.32
115.85
100.76
100.50
77.16 CDCl3
51.43
51.25
47.07
46.90
31.23
30.70
29.97
28.64
60 155 150 145 140 135 130 125 120 115 110 105 100 95 90 85 80 75 70 65 60 55 50 45 40 35 30 25 20 15 10
f1 (ppm)

Methyl 5-(oxiran-2-yl)pentanoate (4)

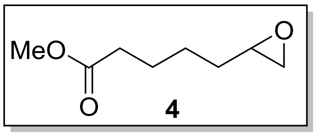

The first step of this reaction is identical to the synthesis of *(E)*-methyl 5-(oxiran-2-yl)pent-2-enoate **2**. The crude mixture was transferred into a 22 mL high pressure reactor. The reactor was pressurised to 20 bar of H_2 and heated at 50 °C for 17 h. Careful solvent evaporation followed by distillation under vacuum provided the desired product **4** as a colourless oil in 53 % overall yield (0.17 g) for the two steps.

For an earlier synthesis of **4** involving alkene epoxidation, see: Muggee, J.; Vogl, O. *J. Polym. Sci, Polym. Chem. Ed.* **1984**, *22*, 2501-2521.

^{1}H NMR (400 MHz, $CDCl_3$): δ = 3.63 (s, 3H, OC*H*$_3$), 2.88 (m, 1H, CH), 2.71 (dd, *J* = 5.0, 4.0 Hz, 1H, C*H*), 2.43 (dd, *J* = 5.0, 2.7 Hz, 1H, C*H*), 2.30 (t, *J* = 7.4 Hz, 2H, C*H*$_2$), 1.70-1.62 (m, 2H, C*H*$_2$), 1.55-1.40 (m, 4H, C*H*$_2$).

^{13}C NMR (100 MHz, $CDCl_3$): δ = 174.1, 52.2, 51.6, 47.1, 34.0, 32.2, 25.6, 24.8.

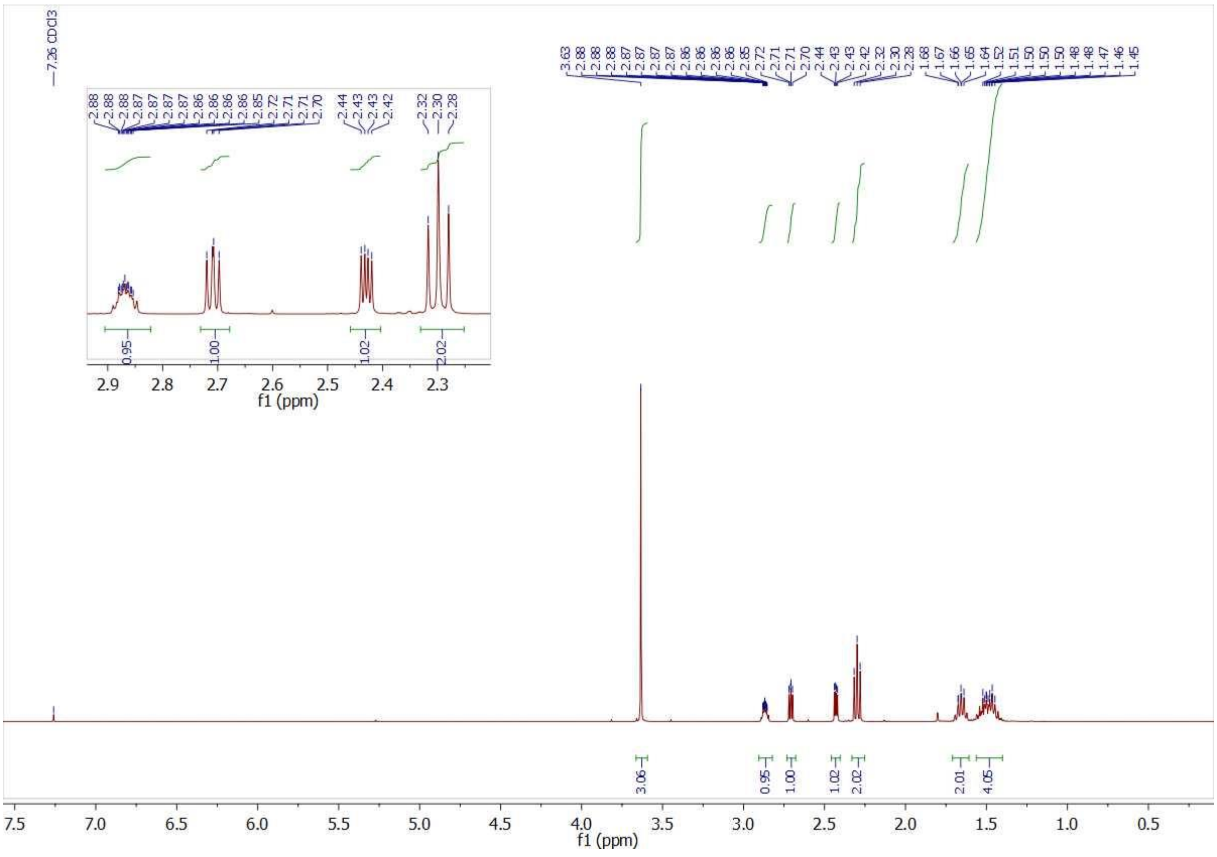

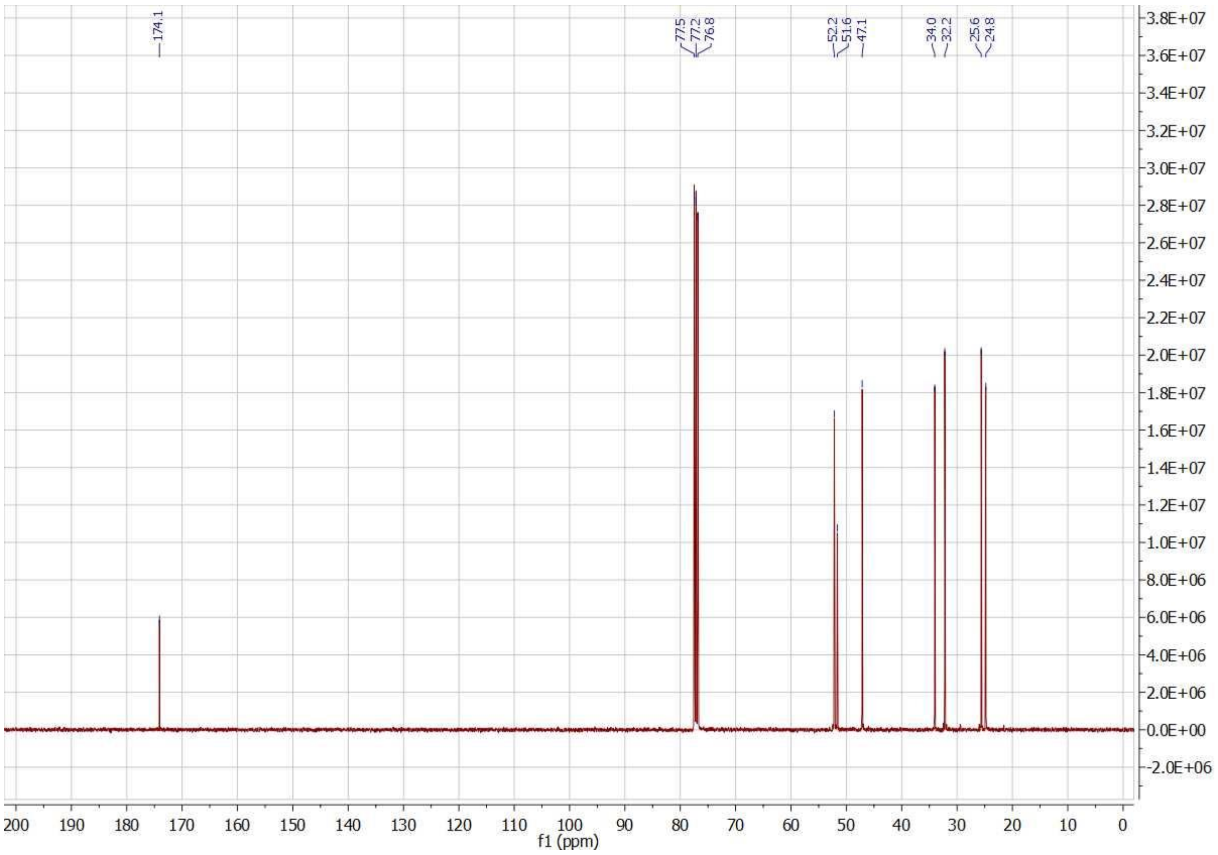

5-(Oxiran-2-yl)pentanenitrile (5)

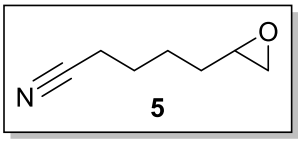

The first step of this reaction is identical to the synthesis of *(E,Z)*-1-cyano-4-(oxiran-2-yl)but-1-ene (**3**). The reaction mixture was concentrated to ~25% of its initial volume and transferred into a high pressure reactor. The reactor was pressurised to 45 bar of H_2 and heated at 50 °C for 17 h. Careful solvent evaporation followed by distillation under vacuum provided the desired product **4** in 46 % overall yield for the two steps.

For earlier syntheses of **5** involving oxorhenium catalyzed alkene epoxidation, see: Yudin, K. A.; Sharpless K. B. J. *Am. Chem. Soc*. **1997**, *119*, 11536-11537. Yudin, K. A.; Chiang, J. P.; Adolfsson, H.; Copéret C. *J. Org. Chem*, **2001**, *66*, 4713-4718.

^{1}H NMR (400 MHz, $CDCl_3$): δ = 2.92-2.85 (m, 1H, C*H*), 2.74 (m, 1H, C*H*), 2.45 (m, 1H, C*H*), 2.35 (t, *J* = 6.9 Hz, 2H, CH_2), 1.78-1.5 (m, 5H, CH_2, C*H*), 1.55-1.43 (m, 1H, CH_2).

^{13}C NMR (100 MHz, $CDCl_3$): δ = 119.6, 51.1, 46.7, 31.6, 25.3, 25.2, 17.1

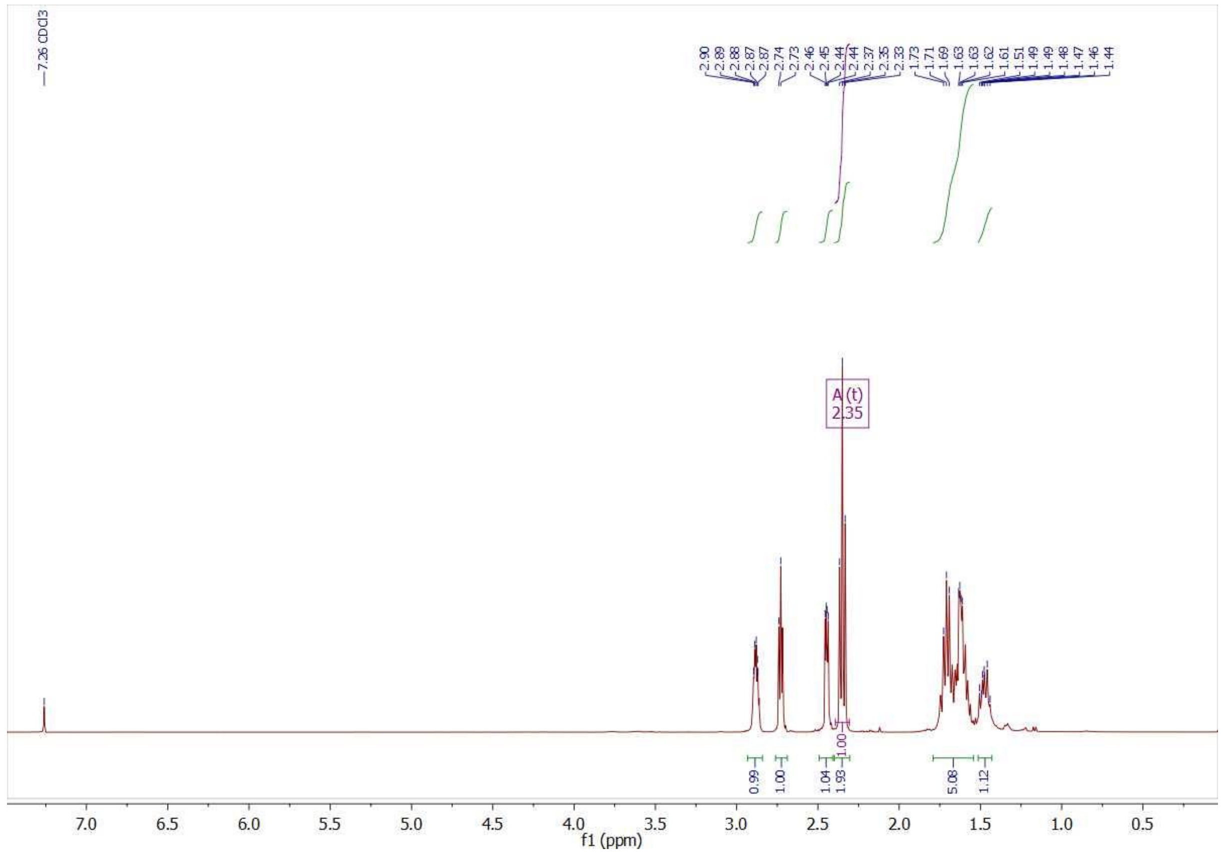
7.26 CDCl3
2.90
2.89
2.88
2.87
2.87
2.74
2.73
2.46
2.45
2.44
2.44
2.37
2.35
2.33
1.73
1.71
1.69
1.63
1.63
1.62
1.61
1.51
1.49
1.48
1.47
1.46
1.44
A (t)
2.35
0.99
1.00
1.04
1.00
1.93
5.08
1.12
7.0
6.5
6.0
5.5
5.0
4.5
4.0
3.5
3.0
2.5
2.0
1.5
1.0
0.5
f1 (ppm)

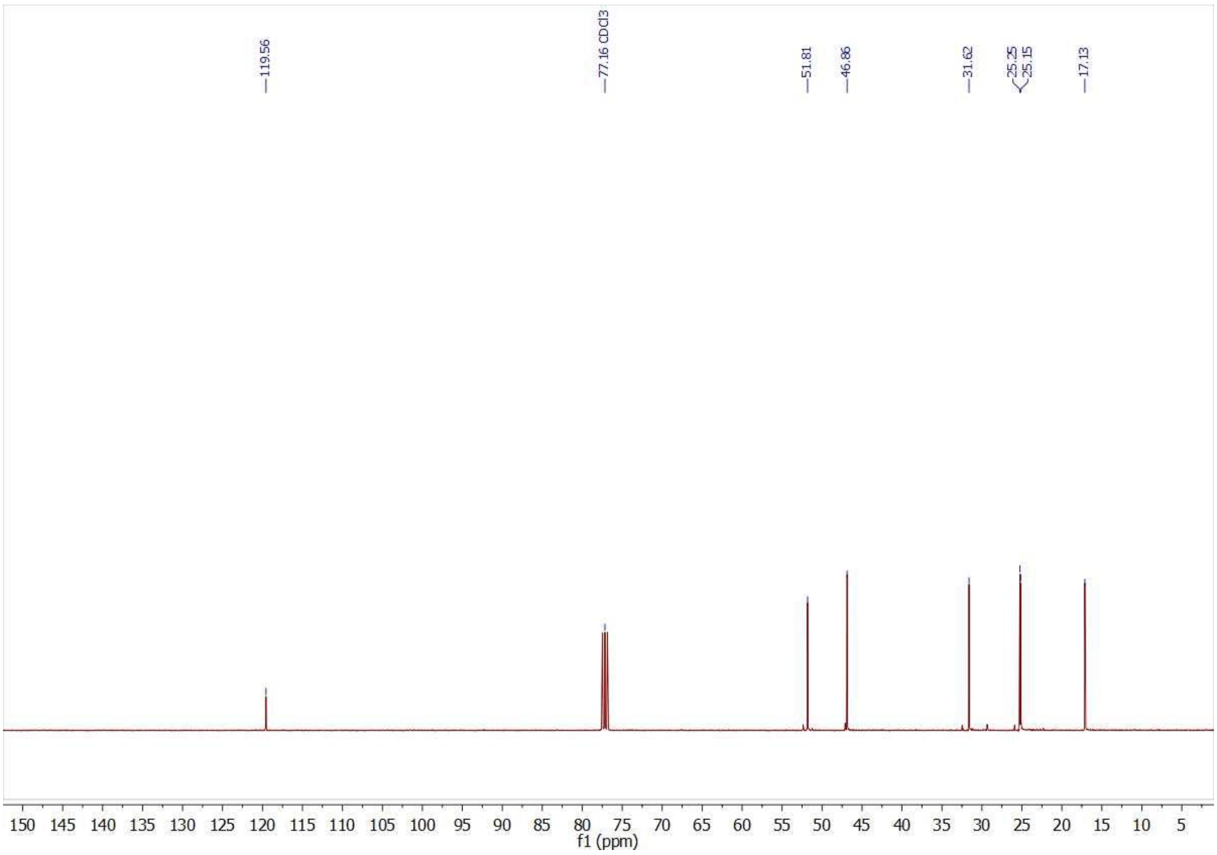

Methyl 6,7-dihydroxyheptanoate (6)

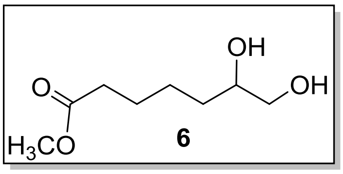

80 mg (0.5 mmol) of **4** and 3 mL of distillated water were introduced in a Schlenk tube and the resulting mixture was stirred at 100 °C for 15 h (full conversion, TLC). The water phase was extracted by 3 × 5 mL of ethyl acetate. The organic phase was dried with Na_2SO_4 and the solvent was evaporated under vacuum to furnish the desired product **6** in quantitative yield. The purity of the product was high enough and no further purification was performed (see NMR data).

^{1}H NMR (400 MHz, $CDCl_3$): δ = 3.75-3.65 (m, 1H, C*H*(OH), 3.66 (s, 3H, OCH_3), 3.62 (dd, *J* = 11.1, 3.0 Hz, 1H, C*H*(OH)), 3.42 (dd, 1H, *J* = 11.1, 7.6 Hz, C*H*(OH)), 2.90-2.50 (m, 2H, OH), 2.32 (t, *J* = 7.3 Hz, 2H, C$H_2$$CO_2$$CH_3$), 1.73-1.57 (m, 2H, C$H_2$), 1.55-1.35 (m, 4H, C$H_2$)

^{13}C NMR (100 MHz, $CDCl_3$): δ = 174.4, 72.1, 66.9, 51.7, 34.0, 32.8, 25.2, 24.9.

HRMS (ESI): [M+Na]+ ($C_8H_{16}O_4Na$); Th: 199.0946, exp: 199.0945

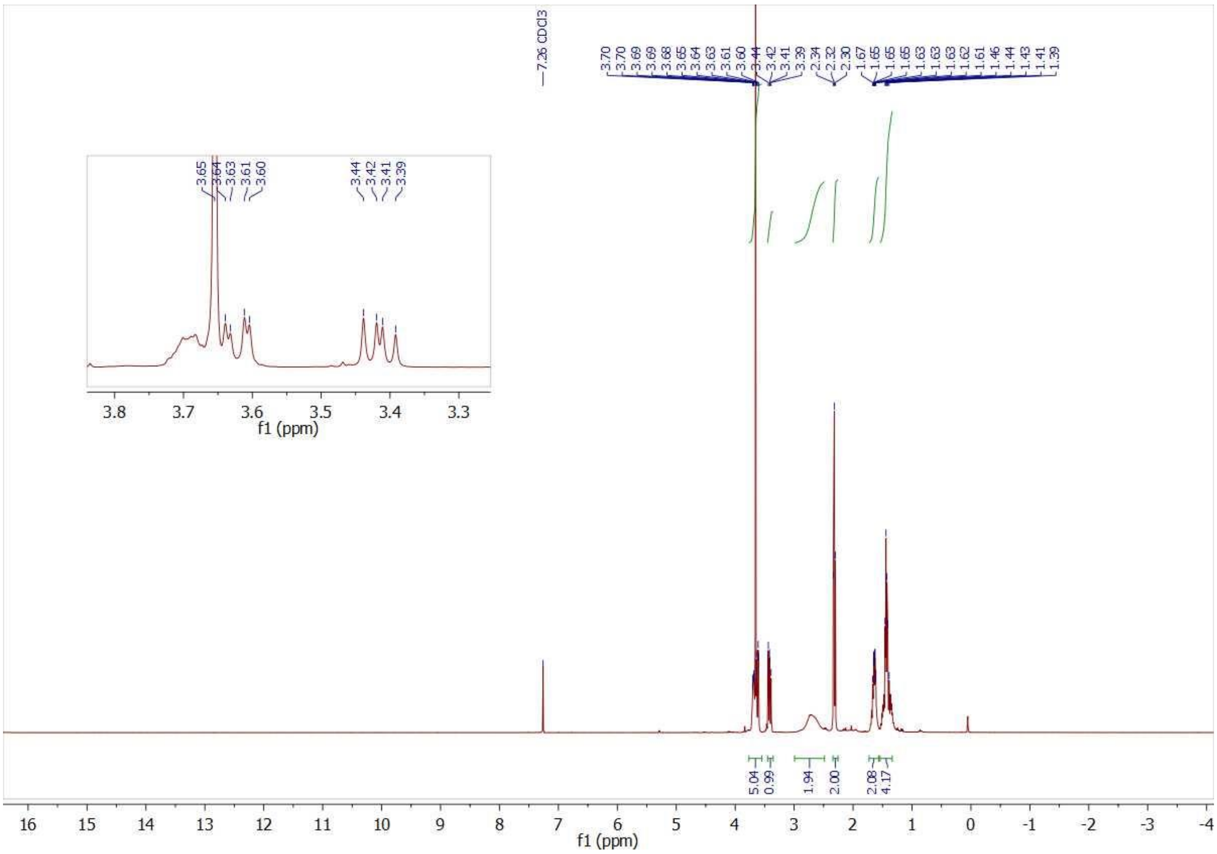

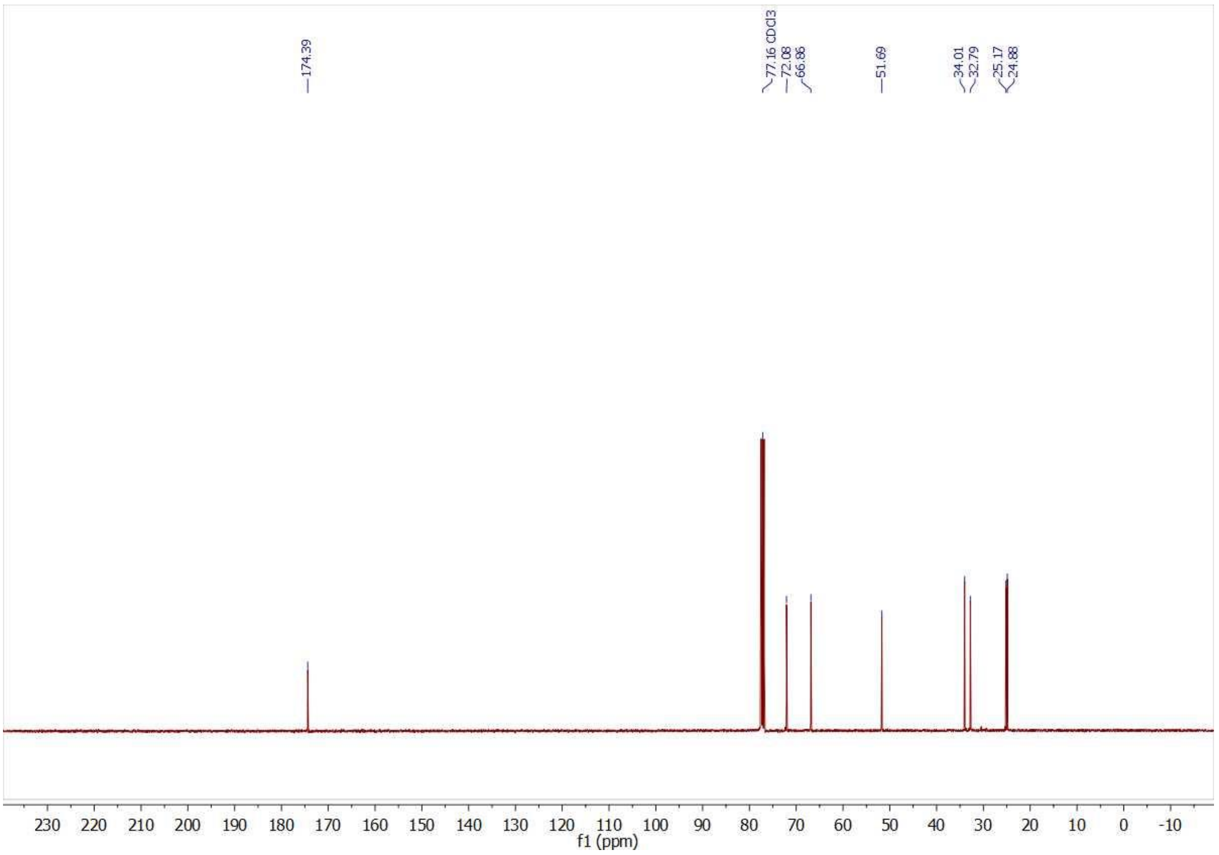

Methyl 6-hydroxy-7-methoxyheptanoate (7)

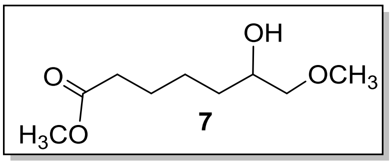

A Schlenk tube was loaded with 158 mg (1 mmol) of **4**, 3 mL of MeOH and 81 mg (1.5 mmol, 1.5 equiv) of MeONa. The reaction mixture was stirred at 65 °C for 17 h. The reaction was quenched by addition of 1 mL of a saturated NH_4Cl. Extraction with 3 × 2 mL of EtOAc and solvent evaporation afforded the desired product **7** as a single regioisomer in a satisfactory purity and quantitative yield.

^{1}H NMR (400 MHz, $CDCl_3$): 3.80-3.72 (m, 1H, C*H*(OH)), 3.66 (s, 3H, OCH_3), 3.41-3.38 (m, 1H, C*H*), 3.38 (s, 3H, CH_3), 3.23 (dd, 9.2 Hz, 8.0 Hz, 1H, C*H*), 2.32 (t, *J* = 7.5 Hz, 2H, CH_2), 1.72-1.60 (m, 2H, CH_2), 1.55-1.35 (m, 4H, CH_2).

^{13} C NMR (100 MHz, MeOD): 175.9, 78.2, 71.0, 59.2, 52.0, 34.7, 34.3, 26,1, 26.1.

HRMS (ESI): [M+Na]+ ($C_9H_{18}O_4Na$); Th: 213.1102, exp: 213.1100

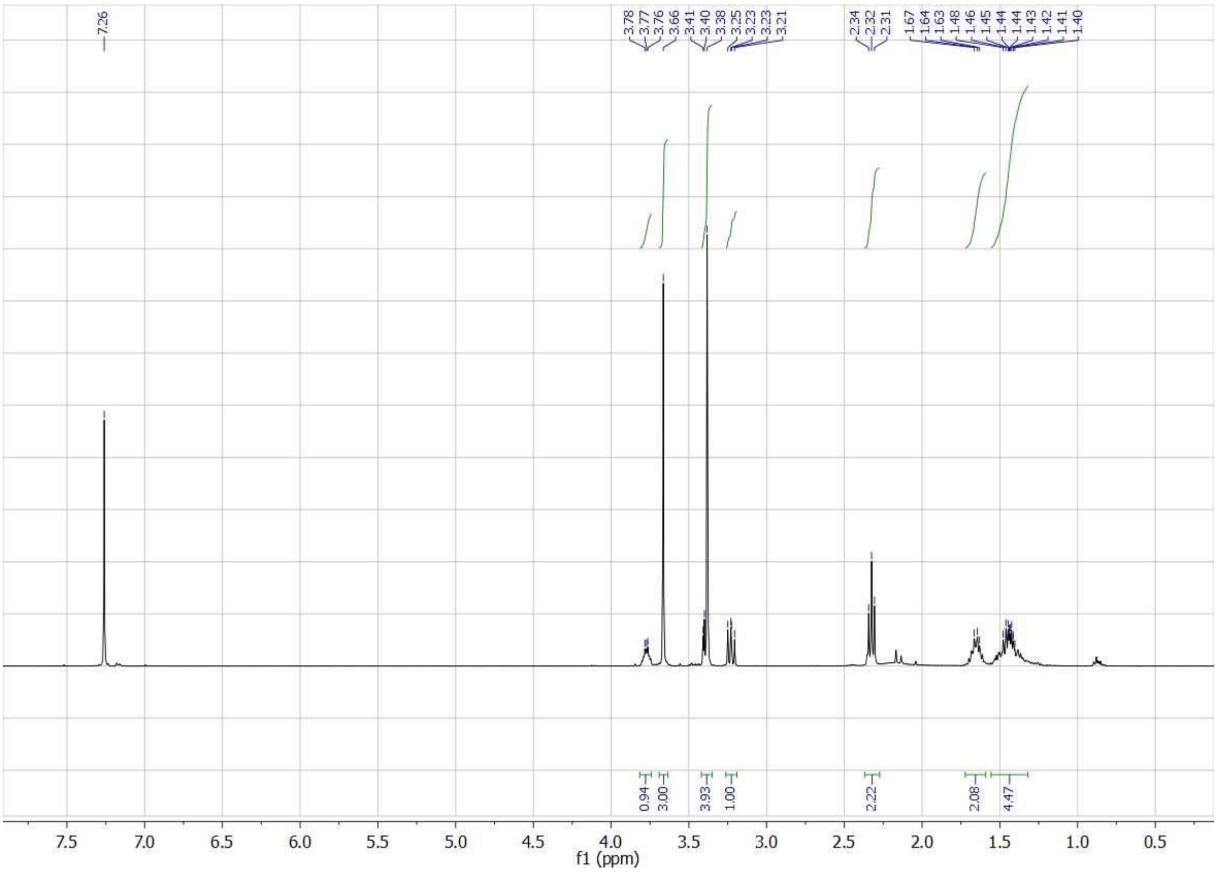
7.26
3.78
3.77
3.76
3.66
3.41
3.40
3.38
3.25
3.23
3.23
3.21
2.34
2.32
2.31
1.67
1.64
1.63
1.48
1.46
1.45
1.44
1.44
1.43
1.42
1.41
1.40
0.94
3.00
3.93
1.00
2.22
2.08
4.47
7.5
7.0
6.5
6.0
5.5
5.0
4.5
4.0
3.5
3.0
2.5
2.0
1.5
1.0
0.5
f1 (ppm)

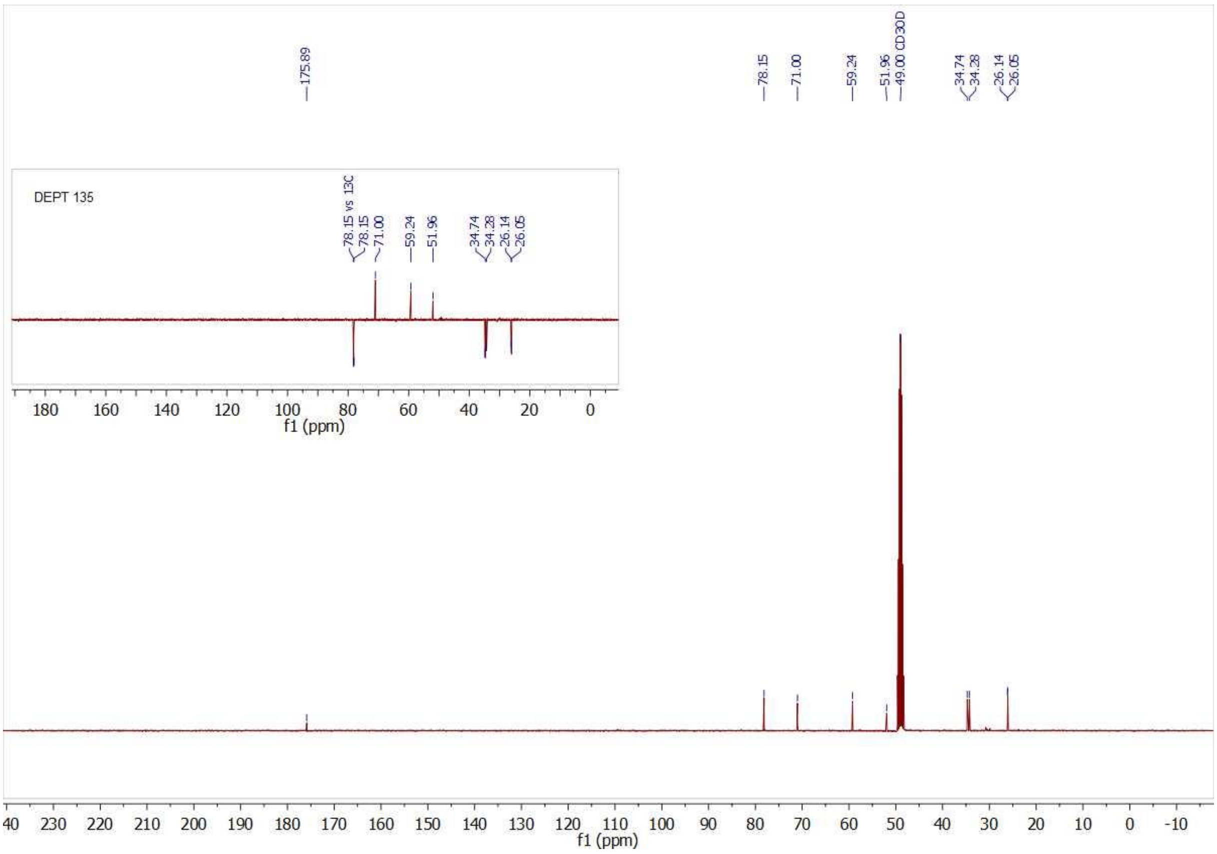
175.89
78.15
71.00
59.24
51.96
49.00 CD3OD
34.74
34.28
26.14
26.05
DEPT 135
78.15 vs 13C
78.15
71.00
59.24
51.96
34.74
34.28
26.14
26.05
180
160
140
120
100
80
60
40
20
0
f1 (ppm)
40
230
220
210
200
190
180
170
160
150
140
130
120
110
100
90
80
70
60
50
40
30
20
10
0
-10
f1 (ppm)

Methyl 6-hydroxy-7-(phenylamino)heptanoate (8)

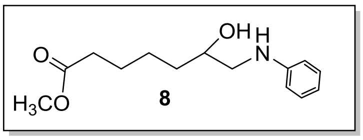

A Schlenk tube was loaded with 158 mg (1 mmol) of **4**, 12 mL of dioxane, 12 mL of water and 0.465 mg (5 mmol, 5 equiv) of aniline. The reaction mixture was heated at 80 °C for 48 h. After solvent removal the crude mixture was purified bu silica gel column chromatography (diethyl ether/petroleum ether 6/4 v/v) to furnish 0.16 g (61%) of **7** obtained as a colourless oil.

^{1}H NMR (400 MHz, $CDCl_3$): 7.19 (dd, *J* = 8.5, 7.4 Hz, 2H, C*H*), 6.75 (tt, *J* = 8.0, 4.0 Hz, 1H, C*H*), 6.67 (d, 8.0 Hz, 2H, C*H*), 3.88-3.81 (m, 1H, C*H*OH), 3.67 (s, 3H, OCH_3), 3.26 (dd, *J* = 12.9, 3.2 Hz, 1H, C*H*), 3.01 (dd, *J* = 12.9, 8.5 Hz, 1H, C*H*), 2.33 (t, *J* = 7.4 Hz, 2H, CH_2CO), 1.75-1.60 (m, 2H, CH_2), 1.60-1.47 (m, 3H, CH_2, CH), 1.47-1.37 (m, 1H, CH).

^{13}C NMR (100 MHz, $CDCl_3$): 174.1, 148.2, 129.3, 117.9, 113.3, 70.0, 51.5, 50.3, 34.6, 33.9, 25.1, 24.8

Elemental analysis ($C_{14}H_{21}NO_3$), Th: C 66.91, H 8.42, N 5.57; Exp C 66.24, H 8.28, 5.32

HRMS (ESI): [M+H]+ ($C_{14}H_{22}NO_3$); Th: 252.1599, exp: 252.1599. [M+Na]+ ($C_{14}H_{21}NO_3Na$); Th: 274.1419, exp: 274.1423

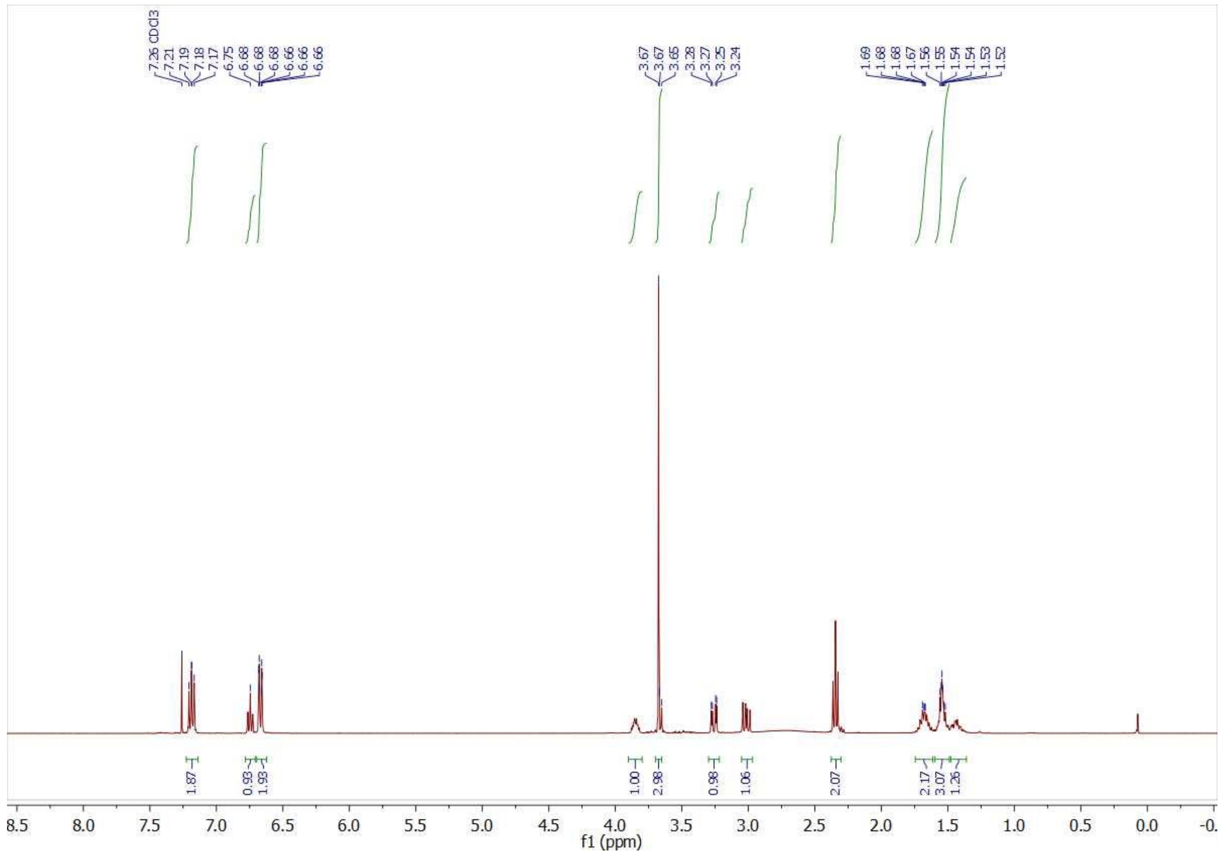
7.26 CDCl3
7.21
7.19
7.18
7.17
6.75
6.68
6.68
6.68
6.66
6.66
6.66
6.66
3.67
3.67
3.65
3.28
3.27
3.25
3.24
1.69
1.68
1.68
1.67
1.56
1.55
1.54
1.54
1.53
1.52
1.87
0.93
1.93
1.00
2.98
0.98
1.06
2.07
2.17
3.07
1.26
f1 (ppm)
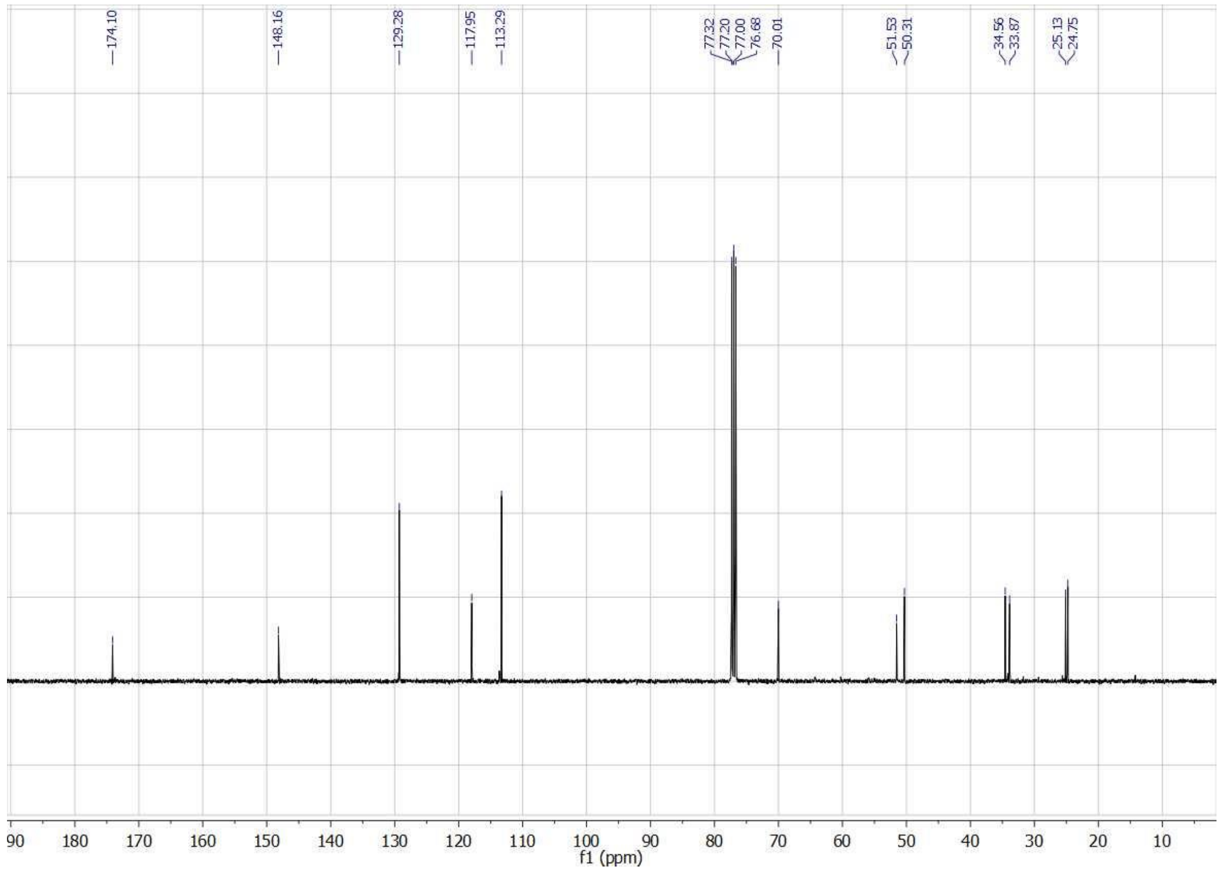
174.10
148.16
129.28
117.95
113.29
77.32
77.20
77.00
76.68
70.01
51.53
50.31
34.56
33.87
25.13
24.75
f1 (ppm)
